# Efficacy and Safety of Intraventricular Thiotepa in Combination With Methotrexate for Leptomeningeal Metastasis From Breast Cancer (NJMU‐BC04): A Single‐Arm Phase II Study

**DOI:** 10.1002/mco2.71014

**Published:** 2026-09-09

**Authors:** Wenbin Zhou, Mengyao Zha, Hehui Fang, Kai Cao, Jiaqi Yong, Yue Sun, Yuan Yuan, Jian Zhang, Shencun Fang, Qiang Ding, Wei Li

**Affiliations:** ^1^ Department of Breast Surgery Department of General Surgery The First Affiliated Hospital with Nanjing Medical University Nanjing China; ^2^ Jiangsu Key Lab of Cancer Biomarkers Prevention and Treatment Jiangsu Collaborative Innovation Center for Cancer Personalized Medicine School of Public Health Nanjing Medical University Nanjing China; ^3^ Department of Oncology The First Affiliated Hospital with Nanjing Medical University Nanjing China; ^4^ Brain Metastases Diagnosis and Treatment Centre The Affiliated Brain Hospital of Nanjing Medical University Nanjing China; ^5^ Department of Oncology Jiangsu Cancer Hospital Nanjing China; ^6^ Department of Oncology Shanghai Medical College Fudan University Shanghai China; ^7^ Phase I Unit Fudan University Shanghai Cancer Center Shanghai China

**Keywords:** intraventricular, leptomeningeal metastasis, methotrexate, thiotepa

## Abstract

Leptomeningeal metastasis (LM), an uncommon yet highly fatal condition, involves malignant tumor infiltration of the meninges resulting in progressive neurological deterioration and severe quality‐of‐life impairment. LM remains largely refractory to treatment and lacks standardized treatment consensus, with a low intracranial objective response rate (iORR) of 20% and a short median intracranial progression‐free survival (iPFS) of 2.2 months. In this study, adult patients with breast cancer‐related LM received intraventricular thiotepa combined with methotrexate. The primary endpoint was achieved with an iORR of 45.8% (11 out of 24, 95% CI: 25.55–67.18%), and the intracranial disease control rate was 58.3% (14 out of 24, 95% CI: 36.64–77.89%). From treatment initiation, the median iPFS and overall survival were 7.9 months (95% CI: 5.3–not estimable) and 11.7 months (95% CI: 5.7–not estimable), respectively. Grade 3–4 treatment‐related adverse events occurred in 62.5% (15 out of 24) of patients, most commonly thrombocytopenia (37.5%) and leukopenia (33.3%), with no Grade 5 toxicities. The exploratory study indicated that two proteins in CSF and four plasma proteins were significantly associated with iORR. Overall, the regimen was feasible and demonstrated a manageable safety profile, with a preliminary signal of clinical benefit warranting further investigation.

## Graphical abstract

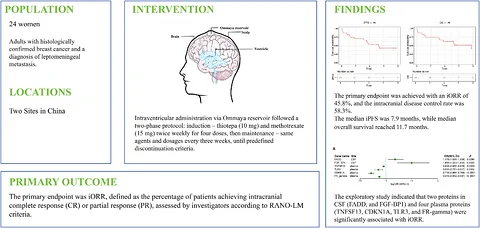

## 1 Introduction

Leptomeningeal metastasis (LM), also termed neoplastic meningitis or leptomeningeal carcinomatosis, is a devastating complication of advanced malignancy characterized by dissemination of tumor cells within the leptomeninges and cerebrospinal fluid (CSF). Although relatively uncommon [1, 2, 3], LM is associated with rapid neurological deterioration, marked impairment in quality of life, and a dismal prognosis, with a median overall survival (OS) of approximately 4 months [4]. Despite advances in systemic anticancer therapy, effective treatment options for LM remain limited, largely because the blood–brain barrier and blood–CSF barrier restrict drug penetration into the central nervous system (CNS) [5, 6]. As a result, current management remains largely palliative and mainly includes radiotherapy, intrathecal chemotherapy, and supportive care [7].

Diagnosis of LM remains challenging, as no available modality provides sufficient sensitivity to reliably exclude meningeal involvement. In clinical practice, diagnosis is based on a combination of CSF cytology, magnetic resonance imaging (MRI), and compatible clinical features in the setting of a known primary malignancy. The EANO–ESMO classification defines LM from solid tumors as Type I (positive CSF cytology or tissue biopsy) or Type II (typical clinical and radiological findings without positive cytology/biopsy) [8]. Systemic drugs poorly penetrate the blood–CSF barrier, limiting therapeutic CSF concentrations. Intrathecal administration bypasses this barrier, delivering drugs directly into the CSF to achieve high local exposure at low systemic doses. Consequently, the ESMO expert consensus recommends intrathecal therapy for most patients with LM who exhibit positive CSF cytology, linear and/or nodular leptomeningeal enhancement on MRI, or a high CSF tumor burden [9]. Intrathecal chemotherapy administration currently encompasses two principal modalities: lumbar puncture and Ommaya reservoir implantation—the latter involving placement of a catheter in the lateral ventricle connected to a subcutaneous reservoir through minimally invasive neurosurgery. For patients who require repeated intrathecal therapy, the Ommaya reservoir is often favored over serial lumbar punctures, as it allows more comfortable administration, more stable CSF drug exposure, a reduced likelihood of epidural puncture‐related complications, and improved intracerebral drug distribution [10, 11, 12, 13, 14, 15, 16]. Prior Phase I prospective trials in lung cancer patients with LM demonstrated that intraventricular pemetrexed administration via the Ommaya reservoir significantly prolonged progression‐free survival (PFS) and OS compared with the lumbar puncture, with a favorable safety profile. Pharmacokinetic studies further revealed that this approach achieved higher CSF drug concentrations and enhanced leptomeningeal tissue penetration, providing a mechanistic basis for its efficacy‐toxicity advantage [17].

However, the efficacy of current intrathecal chemotherapy remains unsatisfactory, with low intracranial objective response rates (iORR) of only about 20% and a median intracranial PFS (iPFS) of 2.2–3.8 months [18]. The two most frequently used intrathecal drugs—methotrexate (MTX) and cytarabine—are cell cycle phase‐specific agents with short CSF half‐lives [13, 19, 20, 21, 22, 23, 24]. Thiotepa, a non‐cell cycle‐specific alkylating agent, presents distinct mechanistic advantages but shares rapid CSF clearance [25, 26]. Despite this shared pharmacokinetic limitation, retrospective studies indicate that thiotepa retains clinical utility [27, 28]; however, it has not demonstrated a significant absolute survival benefit for patients. Intrathecal monotherapy has been employed for decades, yet it exhibits a prominent therapeutic ceiling in the treatment of LM. Systemic combination chemotherapy leverages complementary mechanisms and nonoverlapping toxicities to overcome drug resistance. However, for LM, a randomized controlled trial has shown that the combined intrathecal administration of MTX and cytarabine is not superior to MTX monotherapy [29]. A prospective study [30] of an intrathecal quadruple regimen (MTX, cytarabine, thiotepa, hydrocortisone) showed improved CSF clearance over low‐intensity protocols, yet median OS was not extended, and toxicity was comparable. Furthermore, clinical experience underscores this limitation, as evidenced by a case report of a patient with triple‐negative breast cancer (TNBC)‐derived LM whose disease progressed on this combination regimen. Notably, after switching to a regimen of intrathecal MTX plus thiotepa, the same patient achieved a 10‐month disease remission, with death ultimately attributed to extracranial progression [31]. A preclinical study suggests that MTX can sensitize DNA to damage by depleting nucleotide pools, thereby creating a synergistic effect with DNA‐damaging agents such as alkylators [32]. Although mechanistically justified, the MTX–thiotepa combination lacks established clinical efficacy for breast cancer‐related LM and poses potential additive toxicity risks. Nonetheless, considering the above, we hypothesized that this dual‐agent regimen may offer a favorable therapeutic index for these patients.

As a high‐volume center with extensive experience in the management of LM, we initiated this exploratory, feasibility‐focused study, informed by previous clinical research [30, 31] and our institutional practical experience [33, 34, 35] in a patient population with profoundly limited therapeutic options. This Phase II study (NCT06543992) was conducted to evaluate the efficacy and safety of intraventricular thiotepa combined with MTX via Ommaya reservoir in breast cancer patients with LM. Secondary endpoints included iPFS, OS, extracranial PFS, toxicity, and biomarkers of clinical responses.

## 2 Results

### 2.1 Study Population

**FIGURE 1:**
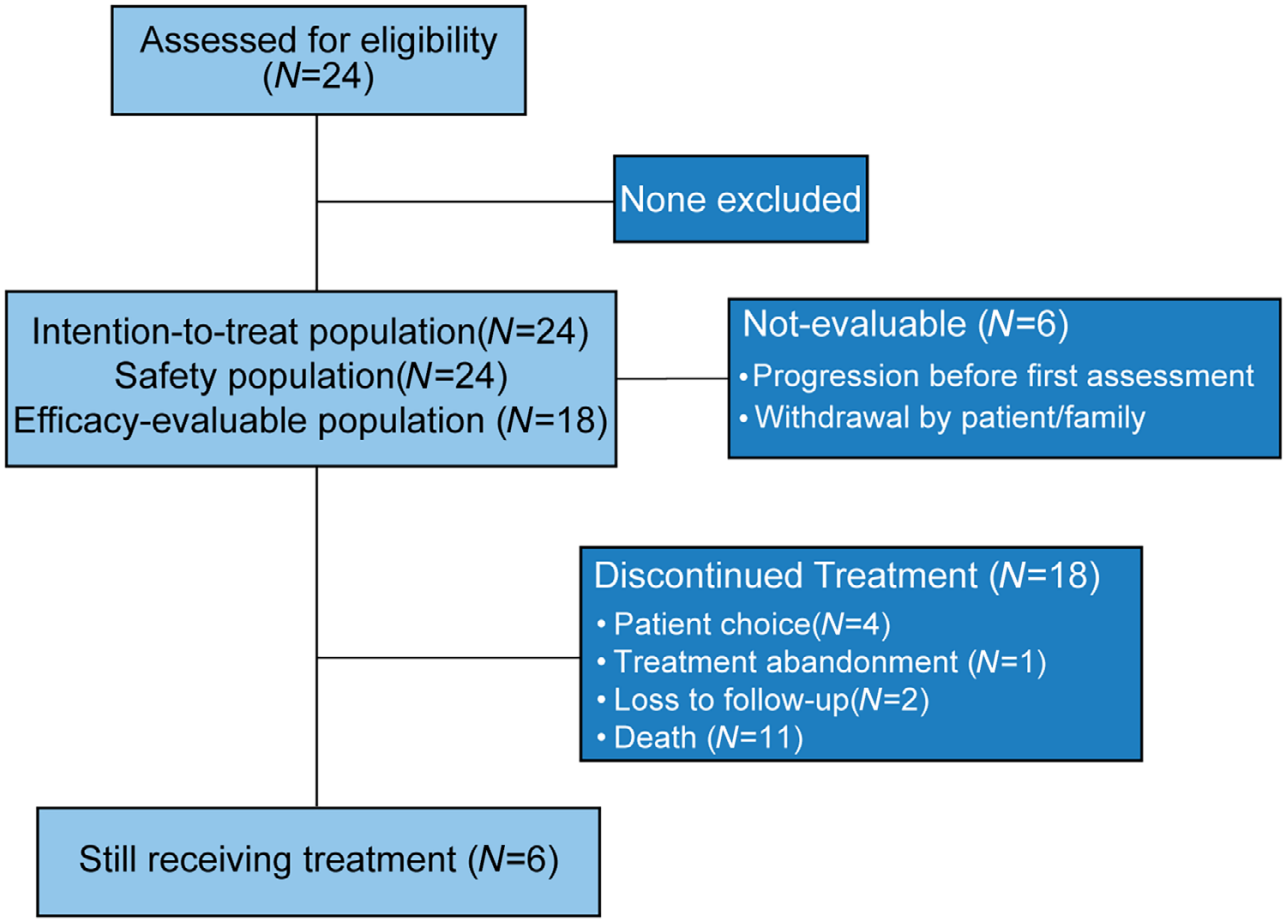
Flow chart of the NJMU‐BC04 study.

From August 2024 to January 2025, 24 participants were enrolled (Figure 1). All 24 patients were recruited from the Jiangsu‐Zhejiang‐Shanghai‐Anhui region of China and were of Han ethnicity. Participant characteristics are summarized in Table 1. The median age of participants was 49 years old (range 33–67) with a median duration of 4 years (range 0–14 years) from initial diagnosis of breast cancer to LM diagnosis. Of these 24 participants, eight were hormone receptor‐positive/HER2‐negative, five were TNBC, and 11 were HER2‐positive. As demonstrated, the majority of participants (83.3%) had an ECOG performance status of 3. Among the 24 participants, 23 (95.8%) exhibited characteristic MRI findings. Sixteen participants (66.7%) had a positive cytology in the CSF at the time of LM diagnosis.

**TABLE 1:** Patients’ characteristics at baseline.

Characteristics	*N* (%) or median (min–max) *N* = 24
BMI	19.5 (14.6–26.7)
Age at enrollment	49 (33–67)
**Stage IV at initial diagnosis**	
Recurrent metastatic disease	22 (91.7%)
De novo metastatic disease	2 (8.33%)
**Molecular subtype**	
HR^−^/HER2^−^	5 (20.8%)
HR^−^/HER2^+^	6 (25.0%)
HR^+^/HER2^−^	8 (33.3%)
HR^+^/HER2^+^	5 (20.8%)
Previous systemic treatments, number of lines	2 (0–10)
**Previous CNS directed therapy**	
No	9 (37.5%)
Yes	15 (62.5%)
**Previous WBRT**	
No	20 (83.3%)
Yes	4 (16.7%)
**Previous stereotactic radiation**	
No	11 (45.8%)
Yes	13 (54.2%)
**Previous intraventricular therapy**	
No	23 (95.83%)
Yes	1 (4.17%)
**Previous surgery for brain metastases**	
No	20 (83.3%)
Yes	4 (16.7%)
**Initial metastasis to the meninges**	
No	21 (87.5%)
Yes	3 (12.5%)
**Non‐LM metastatic sites**	
Bone metastases	12 (50.0%)
Visceral metastases	14 (58.3%)
Brain metastases	17 (70.8%)
Number of metastatic sites at enrollment	3 (0–5)
**Baseline ECOG PS score**	
0	1 (4.17%)
1	0 (0%)
2	3 (12.5%)
3	20 (83.3%)
**LM subtypes**	
Type A: Linear leptomeningeal disease only	14 (58.3%)
Type B: Nodular leptomeningeal disease only	5 (20.8%)
Type C: Both linear and nodular leptomeningeal disease	4 (16.7%)
Type D: Neither linear nor nodular leptomeningeal disease (e.g., no neuroimaging evidence of LM, with possible hydrocephalus only)	1 (4.2%)
**Combined radiotherapy**	
No	15 (62.5%)
Yes	9 (37.5%)
**Combined systemic therapy**	
No	9 (37.5%)
Yes	15 (62.5%)

Abbreviations: HER2, human epidermal growth factor receptor 2; HR, hormone receptor; HR^+^: tumors showing ≥1% of invasive tumor cell nuclei with specific ER and/or PR staining by IHC; HR^−^: tumors showing <1% of invasive tumor cell nuclei with ER and PR expression by IHC; HER2^+^: IHC score 3+, defined as circumferential, complete, intense membrane staining in >10% of invasive tumor cells, or IHC score 2+ (equivocal) with HER2 gene amplification confirmed by ISH; HER2^−^: IHC score 0 or 1+, or IHC score 2+ without HER2 gene amplification by ISH; CNS, central nervous system; WBRT, whole brain radiation therapy; ECOG PS, Eastern Cooperative Oncology Group performance status.

At LM diagnosis, the majority of participants (70.8%) had concomitant intraparenchymal brain metastases. Among these participants, 62.5% had prior central nervous system (CNS) directed therapy. These CNS directed therapies included four participants with prior surgery for brain metastases, four participants with prior WBRT, 13 participants with prior stereotactic radiation. These patients were enrolled due to progression or worsening of CNS metastasis despite prior radiotherapy, indicating limited benefit from previous treatment and unmet clinical needs. Three participants (12.5%) presented with isolated LM without systemic metastases. Before LM occurred, participants had received a median of 3 lines of systemic therapy for advanced disease: chemotherapy (58.3%) and anti‐HER2 therapy combined with chemotherapy (33.3%). Antibody–drug conjugate and endocrine therapy were administered to nine and seven participants, respectively (Table S1). In 50.0% of participants, there were concomitant bone metastatic sites, whereas visceral metastatic sites coexisted in 58.3% of participants. Fifteen participants were on systemic directed therapy and nine participants were treated with radiotherapy during the study.

The predominant presenting symptoms (Table S2) were headache (22 out of 24, 91.7%) and nausea (14 out of 24, 58.3%). Neurological examination at baseline revealed consciousness impairment in eight participants (33.3%). Regarding neurological manifestations, epileptic seizures occurred in six cases (25.0%), sensory deficits in extremities were documented in eight participants (33.3%), motor weakness was identified in 11 individuals (45.8%), and psychiatric disturbances affected eight participants (33.3%). Sensory system manifestations included visual impairment (eight out of 24, 33.3%) and hearing loss (one out of 24, 4.2%). Additional clinical features comprised neuralgia (seven out of 24, 29.2%), dysphagia (eight out of 24, 33.3%), and combined fecal/urinary incontinence (seven out of 24, 29.2%).

### 2.2 Efficacy

**FIGURE 2:**
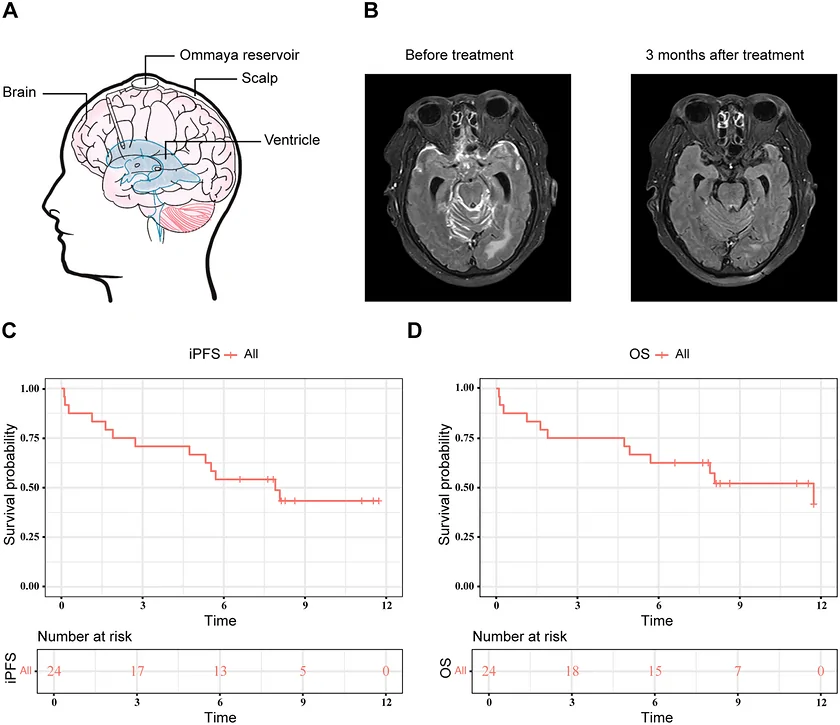
Treatment efficacy. (A) Schematic representation of intraventricular thiotepa combined with methotrexate via Ommaya reservoir; (B) MR imaging before and after treatment; (C and D) Kaplan–Meier curves of iPFS and OS.

The primary endpoint (Table 2 and Figure 2A,B) was achieved with an iORR of 45.8% (11 out of 24, 95% CI: 25.55–67.18%), and the intracranial disease control rate (iDCR) was 58.3% (14 out of 24, 95% CI: 36.64–77.89%) according to the Response Assessment in Neuro‐Oncology Leptomeningeal Metastases (RANO‐LM) criteria. Median iPFS was 7.9 months (95% CI: 5.3–not estimable) (Figure 2C), while median OS reached 11.7 months (95% CI: 5.7–not estimable) (Figure 2D). The upper confidence limit could not be calculated due to limited events beyond the median time and/or heavy censoring in the right tail of the survival curve. At the data cutoff (July 31, 2025), the median follow‐up time was 11.1 months, and six participants (25%) continued receiving treatment in the trial (>6 months' duration: *n* = 6; >9 months' duration: *n* = 2) (Figure S2).

**TABLE 2:** Evaluation of intracranial efficacy.

Characteristics	*N* = 24, *n*(%)
**Neuroimaging assessment**	
PR	7 (29.2%)
SD	8 (33.3%)
PD	3 (12.5%)
Not‐evaluable	6 (25.0%)
**Clinical response evaluation**	
Improvement	10 (41.7%)
Stabilization	5 (20.8%)
Deterioration	3 (12.5%)
Not‐evaluable	6 (25.0%)
**CSF cytological assessment**	
Negative	12 (50.0%)
Positive	6 (25.0%)
Not‐evaluable	6 (25.0%)
**Disease status posttreatment**	
PR	11 (45.8%)
SD	3 (12.5%)
PD	4 (16.7%)
Not‐evaluable	6 (25.0%)

Abbreviations: PD, progressive disease; PR, partial response; SD, stable disease.

Not‐evaluable: The six patients were classified as “not‐evaluable” for the following reasons: they either died due to rapid disease progression before the first scheduled assessment, or treatment was discontinued at the request of the patients and their families.

Fifteen patients received concurrent systemic therapy, including trastuzumab deruxtecan (T‐DXd) (*n* = 9), pyrotinib plus capecitabine (*n* = 1), anlotinib (*n* = 1), afatinib (*n* = 1), eribulin plus bevacizumab (*n* = 1), fulvestrant plus abemaciclib (*n* = 1), and niraparib (*n* = 1). In this subgroup, iORR was 60.0% (nine out of 15, 95% CI: 32.29–83.66%) and iDCR was 73.3% (11 out of 15, 95% CI: 44.90–92.21%); median iPFS and OS were not estimable. Among the nine patients who did not receive concurrent systemic therapy, the iORR was 22.2% (two out of nine, 95% CI: 2.81–60.01%), and the iDCR was 33.3% (three out of nine, 95% CI: 7.49–70.07%). Nine patients received concomitant radiotherapy: seven underwent WBRT, including one with additional focal spinal irradiation, while two received focal intracranial or focal spinal radiotherapy, respectively. In this cohort, iORR was 33.3% (three out nine, 95% CI: 7.49–70.07%) and iDCR was 44.4% (four out of nine, 95% CI: 13.70–78.80%). Median iPFS was 5.7 months (95% CI: 5.3–not estimable) and median OS was 12.0 months (95% CI: 5.7–not estimable).

Among patients who did not receive concomitant radiotherapy, the iORR was 53.3% (eight out of 15, 95% CI: 26.59–78.73%), and the iDCR was 66.7% (10 out of 15, 95% CI: 38.38–88.18%). These findings should also be interpreted cautiously because radiotherapy was not assigned randomly and was administered according to clinical indications, including intracranial disease burden and symptom status.

Neurological symptom evaluation revealed significant clinical improvement in 10 out of 24 participants (41.7%), disease stabilization in five out of 24 (20.8%), and neurological progression in three out of 24 (12.5%). Among the 16 patients with positive CSF cytology at baseline, 10 converted to negative CSF cytology at response assessment, corresponding to a cytologic‐conversion rate of 62.5%. Among the eight patients with negative CSF cytology at baseline, four developed positive CSF cytology during follow‐up. MRI‐based assessments demonstrated partial response (PR) in seven participants (29.2%), stable disease (SD) in eight (33.3%), and radiographic progression in three participants (12.5%).

Systemic disease evaluation per RECIST criteria demonstrated the following therapeutic responses: PR in four participants (16.7%), SD in 13 (54.2%), and progressive disease (PD) in three cases (12.5%) (Table S3).

### 2.3 Safety and Toxicity

All participants who received at least one dose of study therapy were evaluated for toxicity. The safety profile is listed in Table 3. All‐grade treatment‐related adverse events (AEs) occurred in 21 (88%) participants. Grade 3–4 treatment‐related AEs occurred in 15 (63%) participants. The most common AEs were myelosuppression, occurring in 18 participants, with the majority of toxicities categorized as Grade 0–3. No Grade 5 toxicities were observed. Of the 24 participants, the treatment regimen was not changed because of AEs. One participant developed an Ommaya reservoir infection that was managed with antibiotic therapy. Two cases of MTX‐related oral mucositis and a single case of leukoencephalopathy were documented (Table 4).

**TABLE 3:** Treatment‐related adverse reactions.

Toxicity	No event	Grade 1 *n*(%)	Grade 2 *n*(%)	Grade 3 *n*(%)	Grade 4 *n*(%)
Leukopenia	7 (29.2%)	5 (20.8%)	4 (16.7%)	5 (20.8%)	3 (12.5%)
Neutropenia	8 (33.3%)	4 (16.7%)	7 (29.2%)	2(8.33%)	3 (12.5%)
Thrombocytopenia	13 (54.2%)	0	2(8.33%)	5 (20.8%)	4 (16.7%)
Anemia	13 (54.2%)	0	6 (25.0%)	4 (16.7%)	1 (4.17%)
Hepatic impairment	12 (50.0%)	6 (25.0%)	3 (12.5%)	2 (8.33%)	1 (4.17%)
Nausea/vomiting	13 (54.2%)	7 (29.2%)	2 (8.33%)	2 (8.33%)	0
Number of patients	3 (12.5%)	15 (62.5%)	12 (50.0%)	13 (54.2%)	5 (20.8%)

Data are presented as *n* (%). For each toxicity type, patients were classified according to the worst grade observed during treatment, and each patient was counted once per toxicity type. “No event” indicates that the corresponding toxicity was not observed. The summary row indicates the number of patients with at least one treatment‐related adverse event at the corresponding grade; patients with multiple toxicities could contribute to more than one toxicity category.

**TABLE 4:** Treatment‐specific adverse reactions related to intraventricular therapy.

Toxicity	Related to intraventricular therapy (*N*,%)
Oral mucositis	2 (8.33%)
Ommaya reservoir infection	1 (4.17%)
Aseptic meningitis	0(0.00%)
White matter lesion	1 (4.17%)
Mental disorder	1 (4.17%)
Seizure	3 (12.5%)

Within the cohort, 11 deaths were recorded: four were attributed to confirmed meningeal progression, four to intracranial hypertension in the context of leptomeningeal disease, and three to unknown causes. No deaths were identified as directly attributable to the study treatment; however, treatment‐related mortality cannot be completely excluded because of incomplete cause‐of‐death ascertainment. Six patients remain on therapy, with five exhibiting partial responses and one maintaining SD. Seven patients discontinued treatment, citing patient choice (four), treatment abandonment (one), or loss to follow‐up (two).

### 2.4 Biomarkers

**FIGURE 3:**
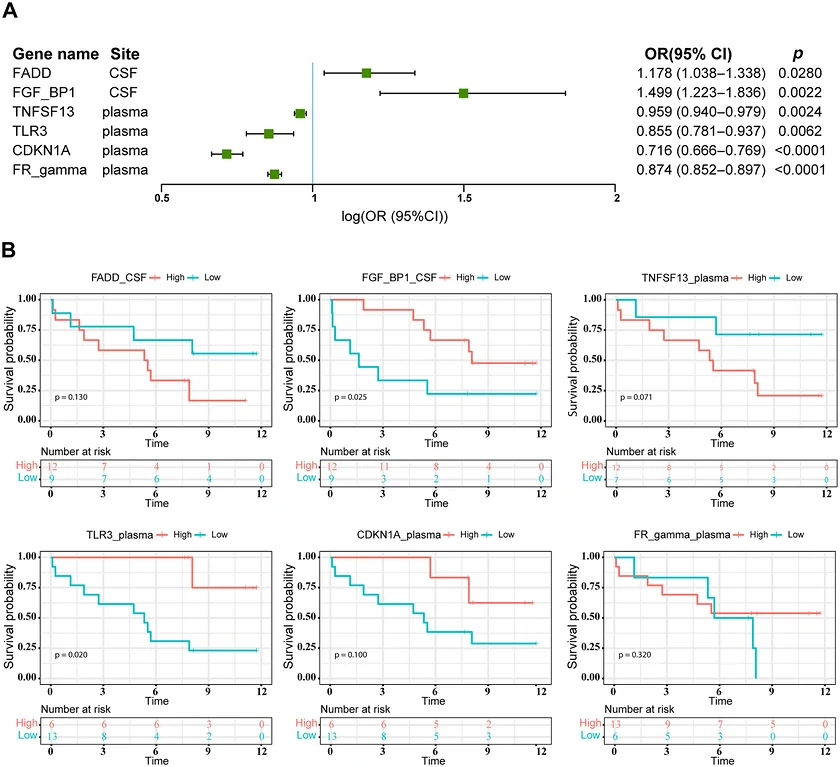
Proteomes biomarkers in CSF and plasma. (A) Multivariate logistic backward stepwise regression indicated two proteins in CSF and four proteins in plasma was associated with iORR. (B) Kaplan–Meier curves showed the predictive value of six proteins of iPFS.

CSF and plasma samples were unavailable from three patients because of insufficient sample volume, and plasma samples were unavailable from two additional patients. Therefore, CSF and plasma samples were available for analysis from 21 and 19 patients, respectively. Clinical data of the whole population and biomarker subgroups are in Table S4. No statistical imputation or specialized methods were applied to handle missing data. Different expression patterns of 92 proteins were found between CSF and plasma (Figure S1). CSF and plasma proteomics (92 proteins) and clinical variables (age, molecular subtypes, and number of previous systemic therapies) were analyzed using LASSO‐penalized logistic regression to identify key features associated with clinical response (iORR). Seven plasma proteins (TNFSF13, hK14, XPNPEP2, CDKN1A, TLR3, PPY, and FR‐gamma) and three proteins (S100A4, FADD, and FGF‐BP1) in CSF were identified. Then, two proteins in CSF (FADD and FGF‐BP1) and four plasma proteins (TNFSF13, CDKN1A, TLR3, and FR‐gamma), significantly associated with iORR (Figure 3A), were found by using multivariate logistic backward stepwise regression.

To further determine the predictive roles of these six proteins in iPFS, the Kaplan–Meier method was applied. High level of FGF‐BP1 in CSF but not FADD was associated with better iPFS (Figure 3B). Moreover, high level of TLR3 in plasma were associated with better iPFS; while the levels of CDKN1A, FR‐gamma and TNFSF13 in plasma were not associated with iPFS (Figure 3B).

## 3 Discussion

MTX and thiotepa have long been established as intrathecal chemotherapeutic agents for LM; however, their historical use as monotherapy has yielded limited clinical benefit [18, 28, 36, 37]. Although isolated case reports have suggested that combination therapy may induce responses lasting up to 10 months [31], prospective investigations of dual‐agent intraventricular regimens remain lacking. Building on the proven success of systemic combination chemotherapy—where complementary mechanisms of action and nonoverlapping toxicity profiles have translated into improved efficacy and mitigation of resistance—we hypothesized that a rationally designed intraventricular combination approach could similarly enhance response rates and prolong response durability in LM.

In 24 enrolled patients, significant prolongation of median iPFS and OS was observed alongside increased iORR, with ongoing responses persisting in a subset. Although intracranial and systemic duration of response were prespecified secondary endpoints, these outcomes were not reported because the limited number of responders, immature follow‐up, and substantial censoring precluded reliable estimation. Historical studies, including the DEPOSEIN Phase III trial [38] of intraventricular liposomal cytarabine administration (iPFS, 3.8 months; mOS, 7.3 months), provide clinical context but are not directly comparable because of differences in eligibility criteria, treatment era, disease burden, and cointerventions. The absence of treatment‐related serious intracranial AEs supports the feasibility of this approach, while its independent therapeutic contribution remains to be defined. The observed therapeutic improvement in this study may be attributed to the following four key design elements: First, the combination of MTX and thiotepa may produce a synergistic antitumor effect. MTX targets actively proliferating cells by inhibiting thymidine synthesis during the S/G1 phase, while thiotepa induces DNA crosslinking with cell cycle‐independent cytotoxicity [39]. Together, the regimen achieves comprehensive coverage across heterogeneous tumor cell populations. Second, compared with the long‐course regimen recommended by the European Association of Neuro‐Oncology/European Society for Medical Oncology guidelines [8], the condensed high‐frequency induction regimen adopted in this study (four administrations over 2 weeks) achieved similar cumulative drug exposure during the critical treatment window, with equivalent dose intensity, while potentially reducing cumulative toxicity and enhancing treatment feasibility. Third, the entire treatment was administered intraventricularly via an Ommaya reservoir, ensuring precise and stable drug delivery. The high proportion of patients with ECOG performance status 3 at baseline likely reflects the substantial neurological symptom burden of leptomeningeal disease, including symptoms related to elevated intracranial pressure, rather than irreversible systemic deterioration alone. In this context, Ommaya reservoir placement may have contributed to symptomatic relief and subsequent functional improvement in selected patients by enabling CSF drainage, pressure management, and intraventricular treatment. Because patients with poor performance status are frequently underrepresented or excluded from clinical trials, the observation of objective responses and disease control in this cohort supports the feasibility of this approach in selected real‐world patients with severe leptomeningeal disease. Given that concomitant systemic therapy and radiotherapy were permitted, the single‐arm design precluded disentangling their effects from those of the intraventricular MTX–thiotepa regimen, and therefore any observed clinical benefit cannot be attributed solely to the intraventricular treatment.

In the subgroup receiving concomitant systemic therapy, intracranial disease control was further improved, with an iORR of 60.0% and an intracranial iDCR of 73.3%; the median iPFS and OS had not been reached at the time of last follow‐up. These findings suggest that systemic therapy—particularly agents capable of penetrating the blood–brain barrier—may enhance intracranial control by reducing overall tumor burden. In this subgroup, some patients (*n* = 9) were treated with T‐DXd. Although the DEBBRAH study [40] reported a median OS of 13.3 months and a median PFS of 8.9 months in patients with HER2‐positive or HER2‐low breast cancer with LM, intracranial efficacy was not specifically evaluated. The consistent benefit observed in our subgroup suggests that the therapeutic effect likely results from multifactorial synergy, including the use of CNS‐active agents and the combined application of systemic and intraventricular therapies. Additionally, the survival outcomes of the concurrent‐therapy subgroup must be interpreted with caution due to inherent guarantee‐time bias. A notable proportion of patients experienced early attrition and therefore did not live long enough to initiate concurrent therapies. As a result, the favorable survival figures observed in this subgroup are likely confounded by this selection bias.

In contrast, the subgroup receiving combined radiotherapy achieved an iORR of 33.3% and an iDCR of 44.4%, with a median iPFS of 5.7 months and a median OS of 12.0 months. The potential synergistic effect of radiotherapy and intraventricular treatment may be attributable to improvements in CSF circulation. Previous studies have shown that approximately 46% of patients with LM develop CSF obstruction [41], and radiotherapy can relieve obstruction in 30–50% of cases [42]. Although radiotherapy has traditionally been regarded primarily as a palliative modality for symptom control [42], targeted radiotherapy strategies have now been incorporated into multiple ongoing clinical trials and clinical guidelines [43].

Molecular subtype analysis indicated that treatment responses varied among patients with different subtypes, with the iORR and iDCR across subgroups summarized as follows: HR^−^/HER2^−^ subgroup, both at 80%; HR^−^/HER2^+^ subgroup, 0% for iORR and 33.3% for iDCR; HR^+^/HER2^−^ subgroup, both at 62.5%; HR^+^/HER2^+^ subgroup, 40% for iORR and 60% for iDCR. Consistent with this observation, prior studies have shown that intraventricular trastuzumab confers clinical benefit in HER2‐positive LM [44, 45, 46]. In a Phase II trial [47], weekly intraventricular trastuzumab (150 mg) resulted in neurological disease stabilization at 8 weeks in 74% of patients, with median LM‐related PFS and OS of 5.9 and 7.9 months, respectively, and acceptable tolerability. Although standardized dosing regimens have yet to be defined, intraventricular trastuzumab remains a relevant therapeutic option for patients with HER2‐positive breast cancer LM. This highlights the importance of developing precise treatment strategies based on molecular profiling for breast cancer patients with LM going forward.

Intraventricular thiotepa combined with MTX exhibited a predictable and largely manageable safety profile, dominated by hematologic toxicity. Myelosuppression occurred in 75% of patients, a frequency consistent with the known systemic toxicity of these agents and likely exacerbated by the heavily pretreated nature of the cohort, including prior chemotherapy and radiotherapy. Importantly, no Grade 5 toxicities or treatment discontinuations were observed, indicating adequate controllability with monitoring and supportive care. Nonhematologic AEs were uncommon; a single case of leukoencephalopathy highlighted the need for neurological surveillance, while device‐related infection and mucositis were manageable. Overall, despite substantial hematologic toxicity, AEs were reversible and did not compromise treatment feasibility, supporting the use of this regimen in specialized centers, with future studies warranted to refine toxicity mitigation strategies.

We identify a bipartite prognostic signature for iORR comprising protective factors—elevated CSF FGF‐BP1 and plasma TLR3. Among six iORR‐associated biomarkers (CSF: FADD, FGF‐BP1; plasma: TNFSF13, CDKN1A, TLR3, FR‐gamma), FADD, CDKN1A, TNFSF13, and FR‐gamma lacked survival significance. Critically, plasma‐based biomarkers demonstrated superior predictive value compared with CSF measurements, while offering the advantage of readily accessible samples that enhance clinical utility.

Fibroblast growth factor binding protein 1 (FGFBP1) is a member of the FGFBP family, which plays a key role in modulating the activity of fibroblast growth factors (FGFs) [48]. Many studies have reported that FGFBP1 is closely related to tumor proliferation, invasion and other biological processes. In TNBC, FGFBP1 promotes tumor proliferation, migration and invasion in vivo and in vitro by activating KLK10–AKT axis [49]. TLR3 plays a crucial role in modulating both innate and adaptive immunity and serves as a critical regulator in cancer, infectious diseases, autoimmune disorders, and allergy [50]. In cancer, TLR3 exhibits a dual role: its expression correlates with both favorable and unfavorable prognoses depending on tumor subtype and microenvironment. The protective role of TLR3 against tumorigenesis may be driven by TLR3 activation of the proapoptotic pathway and intercellular immune interactions [51]. Conversely, TLR3‐mediated protumorigenic mechanisms remain less characterized than their antitumor counterparts; however, emerging evidence implicates TLR3 activation in promoting tumor recurrence and metastatic dissemination [52, 53].

This study has several methodological limitations that warrant cautious interpretation. First, as a single‐arm design without a control group, its findings require confirmation in future randomized controlled trials. Second, the relatively small cohort limits statistical power, precluding robust subgroup analyses and stratification by key clinical variables such as molecular subtype (e.g., HR^−^/HER2^−^, HR^−^/HER2^+^) or prior treatment history (e.g., targeted therapy, radiotherapy, multiline systemic chemotherapy). This constrains the ability to delineate differential efficacy across distinct patient populations and hampers precise application of the results. Third, the causes underlying rapid disease progression in a subset of patients remain unclear and merit further investigation. Fourth, the intraventricular combination regimen used in this study was empirically designed, based primarily on prior studies [30, 31] and our institutional experience [33, 34, 35] rather than on systematic pharmacokinetic or pharmacodynamic modeling. Therefore, the appropriateness of the selected doses and dosing frequency remains to be validated in future prospective studies. Finally, the preliminary nature of the identified proteins, together with the absence of an independent validation cohort, limits the mechanistic interpretation, reproducibility, and generalizability of these findings, which require further validation in larger prospective cohorts and comprehensive mechanistic studies.

The findings of our study further demonstrated that intraventricular thiotepa–MTX combination therapy has potential therapeutic efficacy for breast cancer patients with LM, accompanied by a manageable toxicity profile. Prospective, multicenter randomized trials are essential to validate this approach.

## 4 Materials and Methods

### 4.1 Study Design and Participants

Inclusion criteria required patients to be female, under 75 years of age, with histologically confirmed breast cancer and a new diagnosis of LM, established either by the presence of malignant cells in the CSF or by the combination of characteristic clinical manifestations and MRI findings. Additional eligibility criteria included an Eastern Cooperative Oncology Group (ECOG) performance status of 0–3 and eligibility for Ommaya reservoir placement, either prior to or during the study, to permit intraventricular therapy.

Exclusion criteria included a documented history of other malignancies, severe uncontrolled systemic disorders, known hypersensitivity to thiotepa or MTX, simultaneous participation in other interventional clinical trials, pregnancy, or inability to provide informed consent.

All participants were recruited from The First Affiliated Hospital with Nanjing Medical University and The Affiliated Brain Hospital of Nanjing Medical University.

### 4.2 Procedures

Baseline assessments included a comprehensive clinical evaluation—encompassing weight measurement and determination of ECOG performance status—along with physical and neurological examinations, review of medical history and current medications, and laboratory investigations. Laboratory assessments comprised hematologic parameters, electrolytes, albumin, renal and hepatic function tests, as well as CSF cytological and biochemical analyses. Standardized MRI of the cerebrospinal axis and evaluation of extra‐CNS involvement (via chest, abdominal, and pelvic computed tomography or positron emission tomography scans) were performed concurrently.

Intraventricular administration was carried out using an Ommaya reservoir. The treatment protocol consisted of an induction phase: thiotepa (10 mg) and MTX (15 mg) were administered twice weekly for a total of four consecutive doses, followed by a maintenance phase in which the same agents and dosages were administered every 3 weeks. Treatment was continued until the occurrence of an event meeting predefined discontinuation criteria.

### 4.3 Outcomes

The RANO‐LM Working Group published a standardized consensus on treatment responses for LM [54]. The group recommends incorporating CSF cytology, contrast‐enhanced MRI of the entire neuraxis, and comprehensive neurological assessments using a standardized evaluation form administered at diagnosis and during follow‐up visits. In our study, intracranial treatment response was assessed in accordance with these guidelines. Clinical assessments were scheduled every 21 ± 7 days, and cerebrospinal MRI was performed on the same schedule. All key endpoints, including LM diagnosis, treatment response, and disease progression, were independently reviewed by specialists from three distinct disciplines: neuroradiology (for MRI assessment), neuropathology (for CSF cytology), and neuro‐oncology (for correlation with clinical neurological symptoms). Any discrepancies were discussed and resolved through multidisciplinary consensus meetings.

Treatment‐related toxicity was documented according to NCI‐CTCAE v5.0. Comprehensive physical and neurological examinations, complete blood counts, serum chemistry panels, liver enzyme tests, creatinine measurements, urinalysis, and CSF analyses—including white blood cell and red blood cell counts, glucose, protein, and malignant cell assessment—were performed prior to each therapy cycle, monthly following the completion of treatment, and additionally as clinically indicated.

The primary endpoint was iORR, defined as the percentage of patients achieving intracranial complete response orPR, assessed by investigators according to RANO‐LM criteria. The secondary endpoints included iPFS, OS, toxicity, and biomarkers of clinical responses. OS was calculated from the initiation of therapy to death or the last follow‐up. iPFS was defined as the time from first administration of study treatment to the date of the first objectively documented intracranial disease progression or death from any cause, whichever occurred first.

Blood and CSF samples were analyzed using the Olink Oncology II Panel (Olink Proteomics AB, Uppsala, Sweden), measuring 92 oncology‐related protein biomarkers. Detailed information on these proteins is provided in Table S5. Paired oligonucleotide‐labeled antibody probes bind target proteins, enabling proximity‐driven oligonucleotide hybridization. DNA polymerase addition initiates proximity‐dependent polymerization, generating unique amplifiable sequences. These reporter molecules are subsequently detected and quantified via microfluidic real‐time quantitative polymerase chain reaction systems. The raw results were expressed by normalized protein expression (NPX) values on a log2 scale whereby a higher NPX correlates with higher protein expression.

### 4.4 Statistical Analysis

Previous studies [38] have established a 20% iORR for previous therapy, whereas this combination strategy is expected to achieve 45%. A Simon's optimal two‐stage Phase II design, which minimizes the expected sample size for the trial, was employed with a one‐sided significance level of 0.05 and 80% statistical power. In the first stage, 10 patients would be enrolled, and if three or more objective responses were observed, an additional twelve patients would be enrolled in the second stage. Eight or more responses among the 22 patients during the two stages would be considered that this treatment was active and worthy of further investigation. This design would detect the difference of iORRs between the alternative and null hypotheses. A design inconsistency should be acknowledged: the protocol specified a one‐sided α of 0.025, while the actual study design used a one‐sided α of 0.05. This error arose at the protocol documentation stage.

Patients were assessed for toxicity and included in the final analysis if they received at least one dose of study therapy. Descriptive statistics summarized the cohort, reported as median and range for continuous variables, and as counts and percentages for categorical variables. Median iPFS and OS were estimated using the Kaplan–Meier method. For time‐to‐event endpoints (e.g., iPFS and OS), median estimates are reported along with two‐sided 95% confidence intervals, calculated using the generalized Brookmeyer and Crowley method. For binary endpoints (e.g., ORR and DCR), two‐sided 95% confidence intervals were calculated using the Clopper‐Pearson exact method. To identify potential predictive biomarkers, we fitted a LASSO‐penalized logistic regression model with the regularization parameter (*λ*) selected via 10‐fold cross‐validation. Specifically, we chose the *λ* value corresponding to the minimum mean cross‐validated binomial deviance (lambda.min). Then, we applied a stepwise backward logistic regression to further explore which proteins from CSF or plasma might independently influence the iORR. For survival analyses, protein cutoffs were determined using maximally selected rank statistics, with the minimum proportion (minprop) set at 0.30 to avoid extreme cutoffs. The impact of the protein was presented in forest plots. *p* Values < 0.05 were considered statistically significant.

Safety analyses were conducted on the as‐treated population, excluding patients who did not receive any study therapy. For each AE category, the proportions of patients experiencing any grade of AE, as well as those with severe AEs, were estimated by treatment group.

All statistical analyses were performed by the independent Clinical Data Analysis Department using SAS statistical analysis software (version 9.4; SAS Institute Inc., Cary, NC, USA).

## Author Contributions

Wenbin Zhou, Shencun Fang, Qiang Ding, Jian Zhang, and Wei Li designed and led this study. Wenbin Zhou, Mengyao Zha, and Hehui Fang collected the clinical samples. Mengyao Zha, Kai Cao, Jiaqi Yong, Yue Sun, and Hehui Fang conducted patient follow‐up and data entry. Mengyao Zha, Kai Cao, Jiaqi Yong, Yue Sun, Yuan Yuan, and Hehui Fang performed the statistical analyses. Wenbin Zhou, Mengyao Zha, and Hehui Fang drafted the manuscript. Wenbin Zhou, Shencun Fang, Qiang Ding, Jian Zhang, and Wei Li edited the manuscript. All the authors have read and approved the article.

## Funding

This work was supported by the National Natural Science Foundation of China (grant number 81772475, 82172683 and 82303710), the Natural Science Foundation of Jiangsu Province (grant number BK20230017), Jiangsu Province Capability Improvement Project through Science, Technology and Education (Jiangsu Provincial Medical Key Discipline, grant number ZDXK202222), Jiangsu Province Excellent Postdoctoral Program (grant number 2023ZB006), a project funded by Jiangsu Provincial Science and Technology Department (grant number BE2022807), The JieBangGuaShuai Project for High‐Level Hospital Construction of Jiangsu Province Hospital (grant number CZ1420240211), a project funded by Jiangsu Postgraduate Practice and Innovation Plan (grant number JX10214028), and Beijing Science and Technology Innovation Medical Development Foundation (grant number KC2021‐ZZ‐0010‐3).

## Ethics Statement

This single‐arm/nonrandomized study was reported in accordance with the TREND statement, and the completed checklist is provided as Supporting Information. The study was approved by the ethics committees of The First Affiliated Hospital of Nanjing Medical University (approval No. 2024‐SR‐428) and The Affiliated Brain Hospital of Nanjing Medical University (approval No. 2024‐KYO63‐02) and was registered on ClinicalTrials.gov (NCT06543992). Written informed consent was obtained from all participants. The study was conducted in accordance with good clinical practice guidelines and the Declaration of Helsinki.

## Conflicts of Interest

The authors declare no conflicts of interest.

## Supporting information




**Supporting File 1**: mco271014‐sup‐0001‐SuppMat.docx

## Data Availability

The data generated in this study are available upon request from the corresponding author. Anonymized individual participant data that underlie the reported results can be made available upon request to the corresponding author Dr Wei Li (liwei1218@njmu.edu.cn).
