# Supplementary material for: Efficacy and Safety of Intraventricular Thiotepa in Combination With Methotrexate for Leptomeningeal Metastasis From Breast Cancer (NJMU‐BC04): A Single‐Arm Phase II Study
Source: MedComm (2020). 2026 Sep 9;7(9):e71014. doi: 10.1002/mco2.71014 (PMC13559170; doi:10.1002/mco2.71014)
Supplement: Supplementary file 1 [file MCO2-7-e71014-s001.docx]

**Title page**

**Title:** Efficacy and safety of intraventricular thiotepa in combination with methotrexate for leptomeningeal metastasis from breast cancer (NJMU-BC04): a single-arm Phase II study

**Brief Title**: Intraventricular thiotepa with methotrexate for leptomeningeal metastasis in breast cancer

**Authors:** Wenbin Zhou^1,2#*^, Mengyao Zha^3#^, Hehui Fang^4#^, Kai Cao^3^, Jiaqi Yong^3^, Yue Sun^3^, Yuan Yuan^5^, Jian Zhang^6,7^, Shencun Fang^4*^, Qiang Ding^1,2*^, Wei Li^3*^

**Affiliations:**

^1^ Department of Breast Surgery, Department of General Surgery, The First Affiliated Hospital with Nanjing Medical University, Nanjing, China.

^2^ Jiangsu Key Lab of Cancer Biomarkers, Prevention and Treatment, Jiangsu Collaborative Innovation Center for Cancer Personalized Medicine, School of Public Health, Nanjing Medical University, Nanjing, China.

^3^ Department of Oncology, The First Affiliated Hospital with Nanjing Medical University, Nanjing, China.

^4^ Brain Metastases Diagnosis and Treatment Centre, The Affiliated Brain Hospital of Nanjing Medical University, Nanjing, China.

^5^ Department of Oncology, Jiangsu cancer Hospital, Nanjing, China.

^6^ Department of Oncology, Shanghai Medical College, Fudan University, Shanghai, China

^7^ Phase I Unit, Fudan University Shanghai Cancer Center, No. 270, Dong’an Road, Shanghai 200032, China

^#^These authors contributed equally: Wenbin Zhou, Mengyao Zha, Hehui Fang

***Corresponding Authors:**

Wei Li, PhD, MD liwei1218@njmu.edu.cn

Qiang Ding, PhD, MD [dingqiang@njmu.edu.cn](mailto:dingqiang@njmu.edu.cn)

Shencun Fang, PhD, MD fang1984@njmu.edu.cn

Wenbin Zhou, PhD, MD zhouwenbin@njmu.edu.cn,

**Legends**

**Table S1** Systemic Therapy for Advanced Metastatic Disease Prior to LM

**Table S2** Patients' characteristics of neurological symptoms at baseline

**Table S3** Evaluation of Extracranial Efficacy

**Table S4** Clinical Data of the Whole Population and Biomarker Subgroups

**Table S5** A Comprehensive Overview of the Olink Oncology II Panel

**Figure S1** Heatmap displays the expression levels of proteomes biomarkers in CSF and plasma, with red representing high expression and blue representing low expression

**Figure S2** Survival trajectory plot

**Table S1 Systemic Therapy for Advanced Metastatic Disease Prior to LM**

| ***Characteristics*** | ***N=24, n(%)*** |
| --- | --- |
| Chemotherapy | 14 (58.3%) |
| Combined Anti-HER2 & Chemotherapy | 8 (33.3%) |
| ADC | 9 (37.5%) |
| Endocrine Therapy | 7 (29.2%) |

**Abbreviations:** ADC, Antibody-Drug Conjugate

**Table S2 Patients' characteristics of neurological symptoms at baseline**

| ***Characteristics*** | ***Yes (N, %)*** | ***No (N, %)*** |
| --- | --- | --- |
| Headache/Dizziness | 22 (91.7%) | 2 (8.33%) |
| Nausea/Vomiting | 14 (58.3%) | 10 (41.7%) |
| Visual impairment | 8 (33.3%) | 16 (66.7%) |
| Hypoacusis | 1 (4.17%) | 23 (95.8%) |
| Neuralgia | 7 (29.2%) | 17 (70.8%) |
| Impairment of consciousness | 8 (33.3%) | 16 (66.7%) |
| Dysphagia | 8 (33.3%) | 16 (66.7%) |
| Fecal and urinary incontinence | 7 (29.2%) | 17 (70.8%) |
| Seizure | 6 (25.0%) | 18 (75.0%) |
| Muscle strength abnormality | 11 (45.8%) | 13 (54.2%) |
| Paresthesia of the limbs | 8 (33.3%) | 16 (66.7%) |
| Mental disorder | 8 (33.3%) | 16 (66.7%) |

**Table S3 Evaluation of Extracranial Efficacy**

| ***Characteristics*** | ***N=24, n(%)*** |
| --- | --- |
| CR | 3 (12.5%) |
| PR | 2 (8.3%) |
| SD | 16 (66.7%) |
| PD | 3 (12.5%) |

**Abbreviations:** PD, Progressive disease; SD, Stable disease; PR, Partial response; CR, Complete Response.

**Table S4 Clinical Data of the Whole Population and Biomarker Subgroups**

| ***Characteristics*** | ***Patients with Biomarker Data***  ***N (%) or median (min–max)***  ***N = 21*** | ***All cohort***  ***N (%) or median (min–max)***  ***N = 24*** |
| --- | --- | --- |
| **BMI** | 19.5 (14.6-26.7) | 19.5 (14.6-26.7) |
| **Age at enrollment** | 42(29-65) | 49 (33-67) |
| **Stage IV at initial diagnosis** |  |  |
| recurrent metastatic disease | 19 (90.5%) | 22 (91.7%) |
| de novo metastatic disease | 2 (9.52%) | 2 (8.33%) |
| **Molecular subtype** |  |  |
| HR-/HER2- | 4 (19.0%) | 5 (20.8%) |
| HR-/HER2+ | 6 (28.6%) | 6 (25.0%) |
| HR+/HER2- | 8 (38.1%) | 8 (33.3%) |
| HR+/HER2+ | 3 (14.3%) | 5 (20.8%) |
| **Previous systemic treatments, number of lines** | 2 (0-10) | 2 (0-10) |
| **Previous CNS directed therapy** |  |  |
| No | 8 (38.1%) | 9 (37.5%) |
| Yes | 13 (61.9%) | 15 (62.5%) |
| **Previous WBRT** |  |  |
| No | 17 (81.0%) | 20 (83.3%) |
| Yes | 4 (19.0%) | 4 (16.7%) |
| **Previous stereotactic radiation** |  |  |
| No | 10 (47.6%) | 11 (45.8%) |
| Yes | 11 (52.4%) | 13 (54.2%) |
| **Previous surgery for brain metastases** |  |  |
| No | 17 (81.0%) | 20 (83.3%) |
| Yes | 4 (19.0%) | 4 (16.7%) |
| **Initial metastasis to the meninges** |  |  |
| No | 19 (90.5%) | 21 (87.5%) |
| Yes | 2 (9.52%) | 3 (12.5%) |
| **Non-LM metastatic sites** |  |  |
| Bone metastases | 11 (52.4%) | 12 (50.0%) |
| Visceral metastases | 12 (57.1%) | 14 (58.3%) |
| Brain metastases | 15 (71.4%) | 17 (70.8%) |
| **Number of metastatic sites at enrollment** | 2 (0-5) | 3 (0-5) |
| **Baseline ECOG PS score** |  |  |
| 0 | 0 (0%) | 1 (4.17%) |
| 1 | 0 (0%) | 0 (0%) |
| 2 | 1 (4.76%) | 3 (12.5%) |
| 3 | 20 (95.2%) | 20 (83.3%) |
| **LM subtypes** |  |  |
| Type A: Linear leptomeningeal disease only | 12 (57.1%) | 14 (58.3%) |
| Type B: Nodular leptomeningeal disease only | 4 (19.0%) | 5 (20.8%) |
| Type C: Both linear and nodular leptomeningeal disease | 4 (19.0%) | 4 (16.7%) |
| Type D: Neither linear nor nodular leptomeningeal disease (e.g., no neuroimaging evidence of LM, with possible hydrocephalus only) | 1 (4.76%) | 1 (4.2%) |
| **Combined radiotherapy** |  |  |
| No | 13 (61.9%) | 15 (62.5%) |
| Yes | 8 (38.1%) | 9 (37.5%) |
| **Combined systemic therapy** |  |  |
| No | 8 (38.1%) | 9 (37.5%) |
| Yes | 13 (61.9%) | 15 (62.5%) |
| **Neuroimaging assessment** |  |  |
| PR | 5 (23.8%) | 7 (29.2%) |
| SD | 7 (33.3%) | 8 (33.3%) |
| PD | 3 (14.3%) | 3 (12.5%) |
| Not-evaluable | 6 (28.6%) | 6 (25.0%) |
| **Clinical response evaluation** |  |  |
| Improvement | 7 (33.3%) | 10 (41.7%) |
| Stabilization | 5 (23.8%) | 5 (20.8%) |
| Deterioration | 3 (14.3%) | 3 (12.5%) |
| Not-evaluable | 6 (28.6%) | 6 (25.0%) |
| **CSF cytological assessment** |  |  |
| Negative | 10 (47.6%) | 12 (50.0%) |
| Positive | 5 (23.8%) | 6 (25.0%) |
| Not-evaluable | 6 (28.6%) | 6 (25.0%) |
| **Disease status post - treatment** |  |  |
| PR | 8 (38.1%) | 11 (45.8%) |
| SD | 3 (14.3%) | 3 (12.5%) |
| PD | 4 (19.0%) | 4 (16.7%) |
| Not-evaluable | 6 (28.6%) | 6 (25.0%) |

**Table S5 A Comprehensive Overview of the Olink Oncology II Panel**

| ***UniprotID*** | ***Protein name*** | ***Target 96 Panel*** |
| --- | --- | --- |
| P21589 | 5'-nucleotidase (5'-NT) | ONC II |
| Q8TE58 | A disintegrin and metalloproteinase with thrombospondin motifs 15 (ADAM-TS 15) | ONC II |
| P40222 | Alpha-taxilin (TXLNA) | ONC II |
| P15514 | Amphiregulin (AREG) | ONC II, IRE |
| P04083 | Annexin A1 (ANXA1) | ONC II |
| Q16790 | Carbonic anhydrase IX (CAIX) | ONC II,IMO |
| P16870 | Carboxypeptidase E (CPE) | ONC II |
| P06731 | Carcinoembryonic antigen (CEA) | ONC II |
| P13688 | Carcinoembryonic antigen-related cell adhesion molecule 1 (CEACAM1) | ONC II |
| O60911 | Cathepsin L2 (CTSV) | ONC II |
| O95971 | CD160 antigen (CD160) | ONC II |
| P26842 | CD27 antigen (CD27) | ONC II,IMO |
| P09326 | CD48 antigen (CD48) | ONC II |
| P32970 | CD70 antigen (CD70) | ONC II,IMO |
| Q9UBG3 | Cornulin (CRNN) | ONC II |
| Q9UJ71 | C-type lectin domain family 4 member K (CD207) | ONC II |
| O43927 | C-X-C motif chemokine 13 (CXCL13 ) | ONC II,IMO |
| P38936 | Cyclin-dependent kinase inhibitor 1 (CDKN1A) | ONC II |
| O00548 | Delta-like protein 1 (DLL1) | ONC II |
| P78325 | Disintegrin and metalloproteinase domain-containing protein 8 (ADAM8) | ONC II |
| Q9NQ30 | Endothelial cell-specific molecule 1 (ESM-1) | ONC II |
| P29317 | Ephrin type-A receptor 2 (EPHA2) | ONC II |
| P48023 | Fas antigen ligand (FasL) | ONC II,IMO |
| Q13158 | FAS-associated death domain protein (FADD) | ONC II |
| Q6BAA4 | Fc receptor-like B (FCRLB) | ONC II |
| Q14512 | Fibroblast growth factor-binding protein 1 (FGF-BP1) | ONC II |
| P15328 | Folate receptor alpha (FR-alpha) | ONC II |
| P41439 | Folate receptor gamma (FR-gamma) | ONC II |
| P09958 | Furin (FUR) | ONC II |
| P09382 | Galectin-1 (Gal-1) | ONC II,IMO |
| P35052 | Glypican-1 (GPC1) | ONC II |
| P10144 | Granzyme B (GZMB) | ONC II,IMO |
| P20718 | Granzyme H (GZMH) | ONC II,IMO |
| P14210 | Hepatocyte growth factor (HGF) | INF, ONC II, IMO |
| O75144 | ICOS ligand (ICOSLG) | ONC II,IMO |
| P08069 | Insulin-like growth factor 1 receptor (IGF1R) | ONC II |
| P06756 | Integrin alpha-V (ITGAV) | ONC II |
| P18084 | Integrin beta-5 (ITGB5) | ONC II |
| P15260 | Interferon gamma receptor 1 (IFN-gamma-R1) | ONC II |
| P05231 | Interleukin-6 (IL6) | INF, ONC II, CVD II, IMO, IRE |
| Q9UBX7 | Kallikrein-11 (hK11) | ONC II |
| Q9UKR3 | Kallikrein-13 (KLK13) | ONC II |
| Q9P0G3 | Kallikrein-14 (hK14) | ONC II |
| O60259 | Kallikrein-8 (hK8) | ONC II |
| O95274 | Ly6/PLAUR domain-containing protein 3 (LYPD3) | ONC II |
| Q16674 | Melanoma-derived growth regulatory protein (MIA) | ONC II |
| Q13421 | Mesothelin (MSLN) | ONC II |
| P50579 | Methionine aminopeptidase 2 (MetAP 2) | ONC II |
| Q29983,Q29980 | MHC class I polypeptide-related sequence A/B (MIC-A/B) | ONC II,IMO |
| P21741 | Midkine (MK) | ONC II |
| Q99717 | Mothers against decapentaplegic homolog 5 (MAD homolog 5) | ONC II |
| Q8WXI7 | Mucin-16 (MUC-16) | IMO, ONC II |
| Q96NY8 | Nectin-4 (PVRL4) | ONC II |
| P01298 | Pancreatic prohormone (PPY) | ONC II |
| O00592 | Podocalyxin (PODXL) | ONC II |
| P01133 | Pro-epidermal growth factor (EGF) | ONC II,IMO |
| O00622 | Protein CYR61 (CYR61) | ONC II |
| P31949 | Protein S100-A11 (S100A11) | ONC II |
| P26447 | Protein S100-A4 (S100A4) | ONC II |
| P07949 | Proto-oncogene tyrosine-protein kinase receptor Ret (RET) | ONC II |
| P04626 | Receptor tyrosine-protein kinase erbB-2 (ErbB2/HER2) | ONC II |
| P21860 | Receptor tyrosine-protein kinase erbB-3 (ErbB3/HER3) | ONC II |
| Q15303 | Receptor tyrosine-protein kinase erbB-4 (ErbB4/HER4) | ONC II |
| Q9BXY4 | R-spondin-3 (RSPO3) | ONC II |
| O14828 | Secretory carrier-associated membrane protein 3 (SCAMP3) | ONC II |
| Q9BYH1 | Seizure 6-like protein (SEZ6L) | ONC II |
| P09486 | SPARC (SPARC) | ONC II |
| P21583 | Stem cell factor (SCF) | INF, CVD II,ONC II |
| P18827 | Syndecan-1 (SYND1) | ONC II |
| P56279 | T-cell leukemia / lymphoma protein 1A (TCL1A) | ONC II |
| P37173 | TGF-beta receptor type-2 (TGFR-2) | ONC II |
| P48307 | Tissue factor pathway inhibitor 2 (TFPI-2) | ONC II |
| Q9HBG7 | T-lymphocyte surface antigen Ly-9 (LY9) | ONC II |
| P50591 | TNF-related apoptosis-inducing ligand (TRAIL) | INF, ONC II ,IMO |
| O15455 | Toll-like receptor 3 (TLR3) | ONC II |
| P01135 | Transforming growth factor alpha (TGF-alpha) | INF, ONC II |
| Q14956 | Transmembrane glycoprotein NMB (GPNMB) | ONC II, NEX |
| O75888 | Tumor necrosis factor ligand superfamily member 13 (TNFSF13) | ONC II |
| Q9NS68 | Tumor necrosis factor receptor superfamily member 19 (TNFRSF19) | ONC II |
| P43489 | Tumor necrosis factor receptor superfamily member 4 (TNFRSF4 ) | ONC II,IMO |
| O95407 | Tumor necrosis factor receptor superfamily member 6B (TNFRSF6B) | ONC II |
| P00519 | Tyrosine-protein kinase ABL1 (ABL1) | ONC II |
| P07948 | Tyrosine-protein kinase Lyn (LYN) | ONC II |
| Q14508 | WAP four-disulfide core domain protein 2 (WFDC2) | ONC II |
| P15692 | Vascular endothelial growth factor A (VEGF-A) | INF, ONC II, IMO |
| P35968 | Vascular endothelial growth factor receptor 2 (VEGFR-2) | ONC II,IMO |
| P35916 | Vascular endothelial growth factor receptor 3 (VEGFR-3) | ONC II |
| Q6UXB2 | VEGF-co regulated chemokine 1 (CXL17) | ONC II |
| P08670 | Vimentin (VIM) | ONC II |
| Q9Y5W5 | Wnt inhibitory factor 1 (WIF-1) | ONC II |
| O95388 | WNT1-inducible-signaling pathway protein 1 (WISP-1) | ONC II |
| O43895 | Xaa-Pro aminopeptidase 2 (XPNPEP2) | ONC II |

**Figure S1 Survival trajectory plot**

**Figure S2 Heatmap displays the expression levels of proteomes biomarkers in CSF and plasma, with red representing high expression and blue representing low expression**
